# Bibliometric Analysis and Visualization of Clinical Trials on Psychological Stress and Oral Health (1967-2024)

**DOI:** 10.7759/cureus.57865

**Published:** 2024-04-08

**Authors:** Namrata Dagli, Mainul Haque, Santosh Kumar

**Affiliations:** 1 Karnavati Scientific Research Center (KSRC), School of Dentistry, Karnavati University, Gandhinagar, IND; 2 Pharmacology and Therapeutics, National Defence University of Malaysia, Kuala Lumpur, MYS; 3 Department of Periodontology and Implantology, School of Dentistry, Karnavati University, Gandhinagar, IND

**Keywords:** mental health, dental health, saliva, oral health, scientometric analysis, salivary biomarkers, oral microbiome, psychological stress, network analysis, bibliometric

## Abstract

Stress is ubiquitous in modern life, influencing various facets of human health and well-being. While the impact of stress on mental and physical health is well-documented, its effects on oral health have garnered increasing attention in recent years. This bibliometric analysis explores the literature on the impact of stress on oral health. The study utilizes data from the PubMed database, focusing on publication trends, influential contributors and the temporal analysis of their publications, coauthorship analysis of authors and institutions, key thematic clusters, thematic evolution, and collaboration between various countries. Examining clinical trials investigating the impact of stress on oral health unveils significant trends and insights. Over time, there has been a steady rise in publication frequency, although with occasional fluctuations, indicating an increasing interest in the subject. The University of California has been identified as a leading institution, while Psychoneuroendocrinology emerges as a pivotal journal for disseminating research findings in the field. Keyword analysis reveals diverse thematic clusters, reflecting the multifaceted nature of the impact of stress on oral health. The analysis of topic trends showcases significant shifts over different periods, from basic correlations between mental health conditions and physiological indicators to a broader exploration of psychological interventions and social contexts in recent years. Thematic evolution analysis further elucidates this progression, categorizing themes into motor, basic, niche, and emerging or declining categories. Additionally, the analysis of corresponding authors’ countries uncovers patterns of collaborative efforts, with the United States leading in collaboration levels. In summary, these analyses collectively highlight an evolving comprehension of the impact of stress on oral health, providing valuable insights for clinical practice and guiding future research endeavors.

## Introduction and background

The interplay between psychological stress and oral health has garnered increasing attention in recent years within the field of dentistry and psychology alike. The oral health is intricately linked to an individual’s overall well-being [1-3]. Psychological stress, whether from personal, academic, professional, or societal factors, can profoundly affect oral health, manifesting in various ways within the oral cavity [4,5].

Understanding the complex relationship between psychological stress and oral health is crucial for oral health professionals and psychologists alike. Stress influences oral hygiene behaviors and habits and impacts physiological processes within the oral cavity, such as salivary flow, immune function, and inflammatory responses [4,6,7]. Moreover, psychological stress has been implicated in the development and progression of various oral conditions, including periodontal diseases [8,9], temporomandibular disorders [10], and various oral mucosal lesions [11]. Within the confines of the oral cavity, stress manifests in various ways, ranging from increased susceptibility to oral diseases to the exacerbation of existing conditions. This intricate relationship between psychological well-being and oral health underscores the importance of a holistic approach to dental care that recognizes the interconnectedness of mind and body. Exploring the mechanisms through which stress influences oral health provides valuable insights into preventive and therapeutic approaches. Additionally, recognizing oral manifestations of stress can indicate an individual’s overall psychological well-being [11].

With the ever-increasing volume of published scientific literature, it becomes challenging to review and analyze the existing body of knowledge systematically. Bibliometric analysis, a quantitative method widely used in scientific research, offers a comprehensive approach to assessing the scientific landscape, trends, and patterns within a particular field. By systematically analyzing key research themes, influential authors, leading sources, leading research groups, and collaborative networks, this bibliometric analysis aims to shed light on the current state of knowledge and future research directions on the impact of stress on oral health and ultimately contribute to advancements in both dental and mental health.

## Review

Methodology

Study Selection and Data Collection

The online search was conducted on March 21, 2024, utilizing the PubMed database, a widely recognized repository of biomedical literature. The search strategy was tailored to include articles published in the English language and classified as clinical trials, ensuring the relevance and reliability of the findings. The following search string combining relevant keywords and Medical Subject Headings (MeSH) terms was employed to optimize the retrieval of relevant articles: (psychological stress) AND (“oral health” OR “oral health” OR “oral cavity” OR “oral microbiota” OR “oral diseases” OR “periodontal diseases” OR “dental caries” OR “gingivitis” OR “periodontitis” OR “salivary cortisol” OR “salivary biomarkers” OR “Oral tissues” OR “Oral mucosa”) NOT “mechanical stress” NOT “plant.” The filters were applied in the PubMed database for language and article type. An additional filter, “Duplicate articles” in the PubMed database, was used to check the duplicate articles. Then, the remaining articles were exported to a text file for further analysis. The flowchart of the study selection process was generated according to the Preferred Reporting Items for Systematic Reviews and Meta-Analyses (PRISMA) guidelines [12].

Data Analysis and Visualization

Following the systematic search, retrieved articles were imported into bibliometric analysis software, including VOSviewer version 1.6.20 [13] and Biblioshiny (R Studio version 4.3.1) [14], for further analysis and the relevant metadata from the retrieved articles, including publication title, authors, title, abstract, publication year, type of articles, keywords, and MeSH terms were extracted. Subsequently, bibliometric analysis and visualization were done to understand the co-occurrence patterns among keywords and coauthorship patterns in authors and institutions using VOSviewer. The most relevant contributors based on the number of published clinical trials, temporal analysis of their publications, thematic evolution, topic trends, and collaborations between countries within the retrieved articles were identified and visualized using the Biblioshiny App. Additionally, Microsoft Excel and Biorender [15] were utilized to create graphical representations of the findings, facilitating data interpretation and presentation.

The rigorous application of these methods aimed to provide a comprehensive overview of the scholarly landscape surrounding the impact of stress on oral health. Through systematic analysis and visualization of the literature, this study sought to identify research trends, knowledge gaps, and potential avenues for future investigation in this critical area. The study adhered to ethical guidelines, respected copyright laws in using published materials, and properly attributed the original authors and sources.

Results

**Figure 1:**
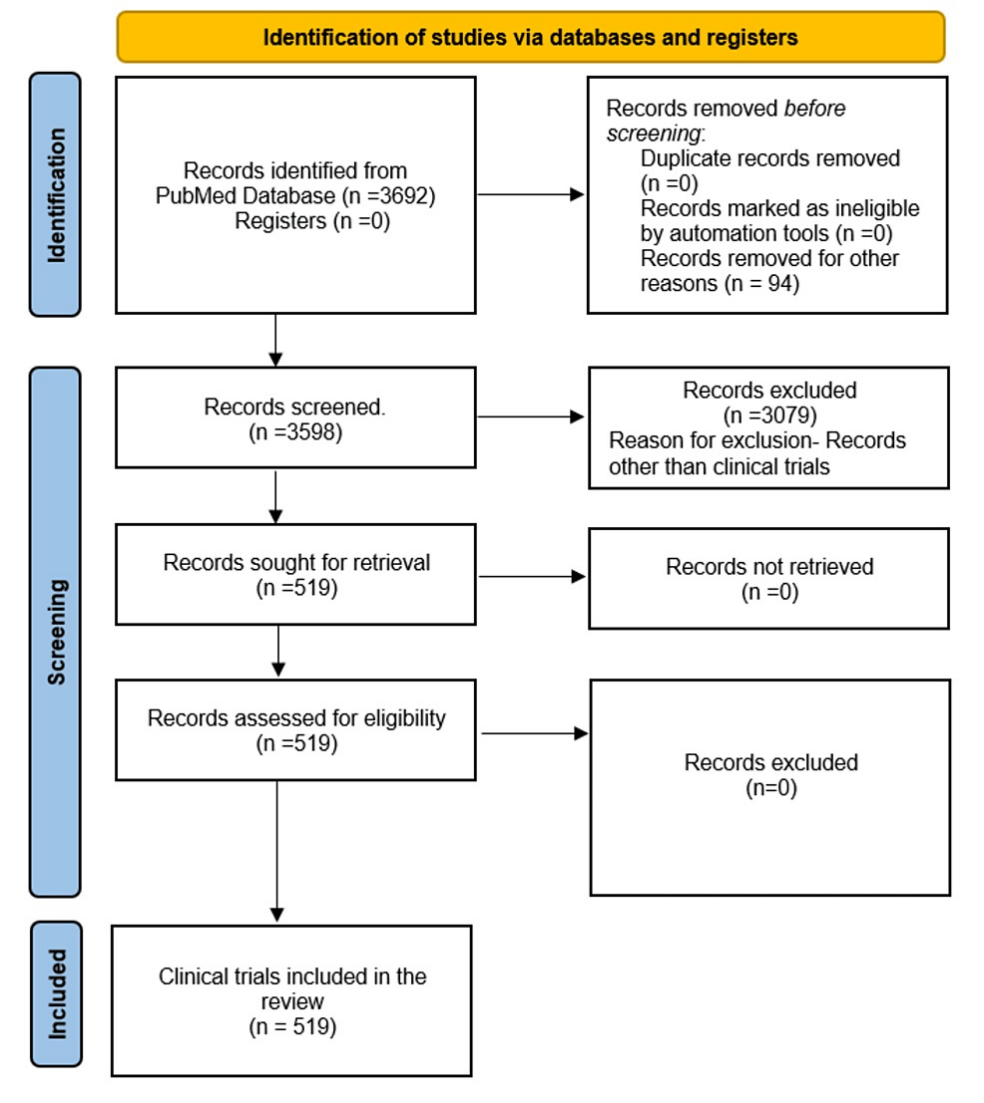
Flowchart depicting the process of selecting studies. Image credit: Namrata Dagli.

Of the 3,692 results in the PubMed database, 3,598 met the English language criteria. This set contained 187 reviews, 28 case reports, 4 editorials, and 10 comments. Additionally, 519 clinical trials were identified (Figure 1). No duplicate publications were found. Biblioshiny software found that the 519 clinical trials published in English involved 2,560 authors and were distributed across 255 sources from 1992 to 2024. In addition, the software analysis revealed that among these authors, only three contributed single-authored documents, while international coauthorship accounted for 9.056%. On average, there were 5.68 coauthors per document, and the annual growth rate was 2.19%. A total of 2,087 keywords were identified.

Publishing Trend of Articles

**Figure 2:**
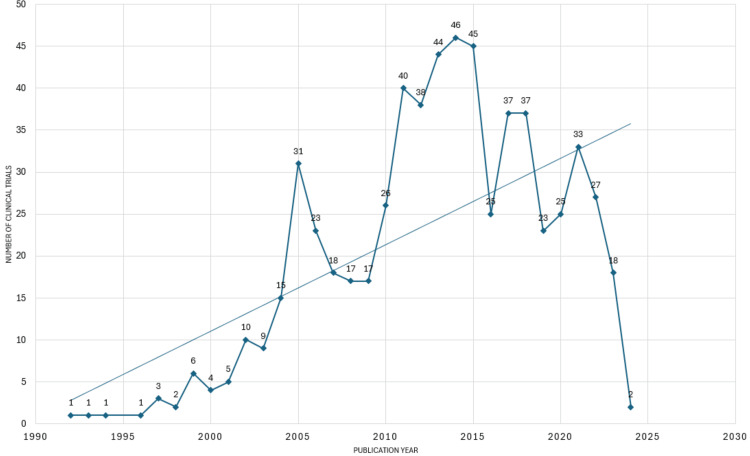
The annual scientific publication of clinical trials on the impact of stress on oral health. Image credit: Namrata Dagli.

Figure 2 illustrates a fluctuating pattern in publication frequency, with an overarching upward trend. High publication rates during 2013-2015 suggest increased activity or interest in clinical trial research during those years. The surge in publications between 2004 and 2005 indicates a significant growth or momentum in clinical trials on the topic. Conversely, the sharp decline between 2015 and 2016 suggests a sudden drop-off in published trials, which could reflect various factors, such as shifts in funding, regulatory changes, or research priorities. Moreover, the noticeable decrease in publications post-2021 indicates a potential slowdown or decline in clinical trial activity in recent years.

Most Relevant Authors

**Figure 3:**
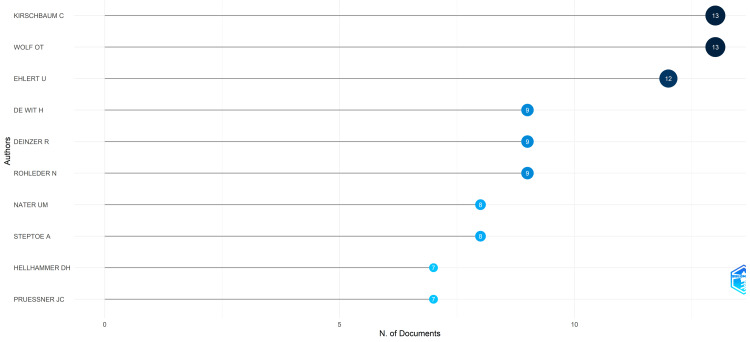
The most relevant authors based on the number of published clinical trials in the PubMed database on the impact of stress on oral health. Image credit: Namrata Dagli.

The authors Kirschbaum C and Wolf OT emerge as the most prominent authors in terms of number of published clinical trials, closely trailed by Ehlert U. Collectively, the 10 most relevant authors account for 18.3% of the total clinical trials published on the topic in PubMed. Interestingly, the top two authors alone, Kirschbaum C and Wolf OT, contribute 27.37% of the papers produced by this group. This data is illustrated in Figure 3.

**Figure 4:**
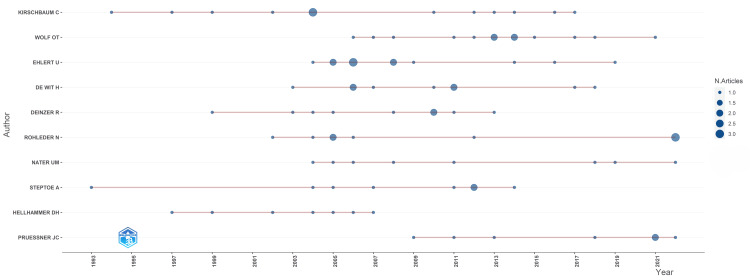
Author’s production over time. Image credit: Namrata Dagli.

Figure 4 showcases the productivity trends of these influential authors over time, specifically focusing on their publication output. It is evident that the peak period of productivity spans from 2004 to 2014.

Coauthorship Analysis of Authors

**Figure 5:**
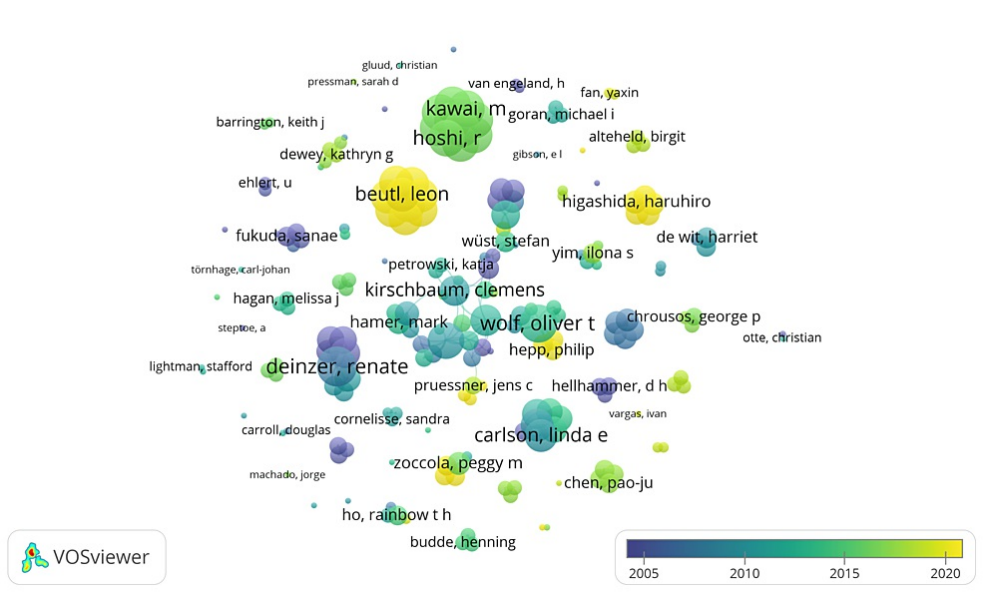
Overlay visualization of coauthorship of authors. Weight: total link strength. Scores: average publication year. Notes: The size of the nodes represents the number of published clinical trials on the impact of stress on oral health. Image credit: Namrata Dagli.

We identified 2,625 authors in the PubMed database, each with at least one published clinical trial on the impact of stress on oral health. When we set the threshold to a minimum of two documents, we found 202 authors. Using VOSviewer, we calculated the total strength of coauthorship links for each author and visualized the collaboration network of these 202 authors in an Overlay visualization. In this visualization, nodes represent authors, and connections between nodes indicate collaborations. Node size corresponds to the total link strength (TLS), while node color reflects the average publication year of the authors’ documents. Our analysis uncovered 62 clusters among these 202 authors, comprising 309 links, with a TLS of 579 (Figure 5). Notably, Renate Deinzer had the highest TLS of 21, with eight published clinical trials, followed by Oliver T Wolf with a TLS of 19 and 13 published clinical trials.

Most Relevant Institutions or Universities and Their Productivity Over Time

Table 1 presents the most relevant institutions or universities based on the number of clinical trials published regarding the impact of stress on oral health. Combined, these five leading institutions contributed to 31.8% of all publications in the PubMed database on this topic. Specifically, the University of California alone accounted for 13.5% of these publications.

**Table 1:** Most relevant universities based on the volume of clinical trials published regarding the impact of stress on oral health in the PubMed database.

Institutions	Number of clinical trials
University of California, United States	70
University of Trier, Germany	28
University of Regensburg, Germany	24
University of Vienna, Austria	22
Technische Universität Dresden, Germany	21

**Figure 6:**
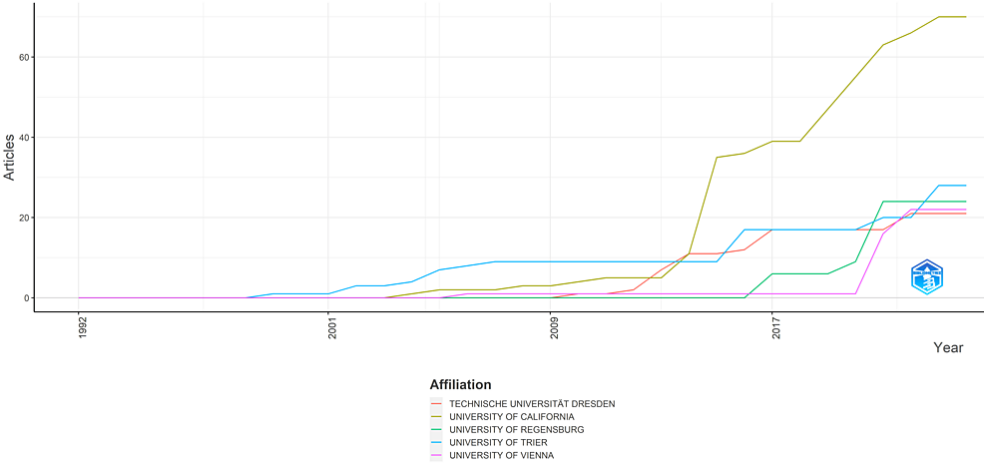
Production of universities over time. Image credit: Namrata Dagli.

Figure 6 illustrates the temporal trend of clinical trials published in the PubMed database over time. The most significant increase is evident in the volume of publications from the University of California from 2014 to 2015. Before this surge, the University of Regensburg held the title of the most productive university from 1998 to 2013, after which the University of California assumed the leading position.

Coauthorship Analysis of Institutions or Universities

**Figure 7:**
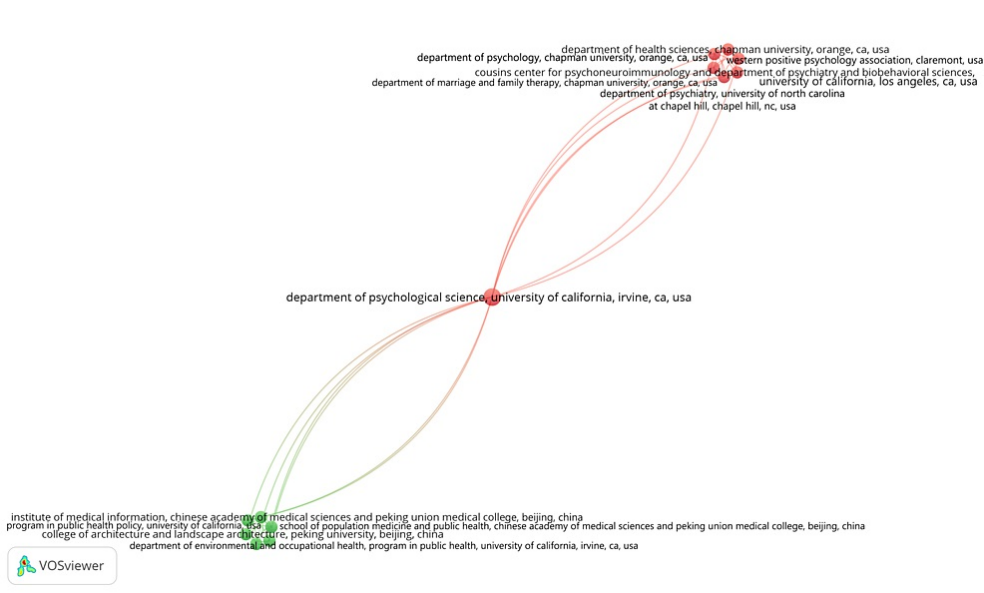
Network visualization of coauthorship analysis of the largest set of connected institutions. Weight: Documents. Image credit: Namrata Dagli.

A total of 1,155 institutions or universities were identified, each having at least one published clinical trial on the impact of stress on oral health in the PubMed database. When the threshold of a minimum number of documents was set to two, only 21 institutions or universities met the criteria. Using VOSviewer, the total strength of coauthorship links with other institutions or universities was calculated for all 1,155 institutions. The institutions with the highest link strengths are presented in Table 2. The largest set of connected items included 13 institutions grouped under two clusters with 42 links. Cluster 1 is depicted in red and Cluster 2 in green (Figure 7).

**Table 2:** Universities with the highest total link strength. Notes: The total link strength value reflects the intensity of collaboration between the universities based on the number of coauthored documents.

University	Number of publications	Total link strength
Department of Biomedical Sciences, University of Veterinary Medicine, Vienna, Austria	2	13
Department of Child and Adolescent Psychiatry, Medical University of Vienna, Austria	2	13
Department of Clinical and Health Psychology, Faculty of Psychology, University of Vienna, Austria	2	13
Department of Pediatrics and Adolescent Medicine, Medical University of Vienna, Austria	2	13
Outpatient Unit for Research, Teaching and Practice, Faculty of Psychology, University of Vienna, Austria	2	13
Working Group Entertainment Computing, University of Vienna, Austria	2	13

Most Relevant Journals

Table 3 presents the sources with the highest number of published clinical trials regarding the impact of stress on oral health. These most relevant sources collectively contributed to 28.7% of all publications in this field. Particularly noteworthy is Psychoneuroendocrinology, which emerged as the most influential source, accounting for 18.3% of the clinical trials published in the PubMed database in this domain.

**Table 3:** The most relevant sources based on the number of clinical trials published in the PubMed database on the impact of stress on oral health.

Journals	Number of clinical trials
Psychoneuroendocrinology	95
Stress (Amsterdam, the Netherlands)	16
PLoS One	15
Psychosomatic Medicine	9
Hormones and Behavior	7
Psychopharmacology	7

**Figure 8:**
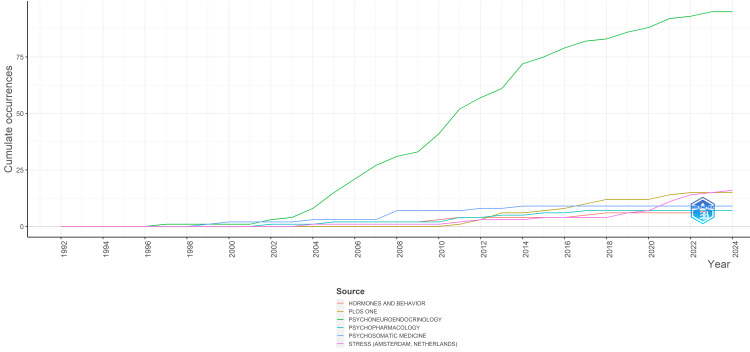
Journals’ production over time. Notes: The Y axis indicates the cumulative number of published clinical trials. Image credit: Namrata Dagli.

Figure 8 illustrates the temporal trend of published clinical trials in these prominent sources over time. There has been a noticeable uptick in the volume of publications in Psychoneuroendocrinology since 2003 and continuing to the present (Figure 8).

Keywords Analysis

**Figure 9:**
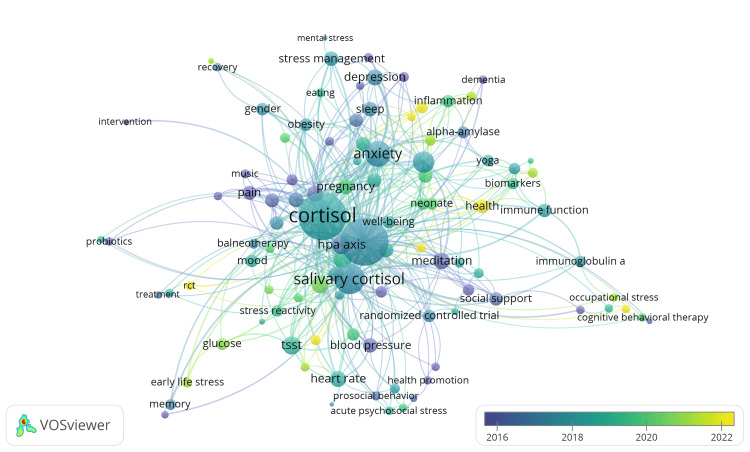
Overlay visualization of co-occurrence of keywords. Notes: The size of the node indicates the total link strength value while the thickness of connecting lines indicates the strength of the co-occurrence relationship between keywords. Weight: Total link strength. Scores: Average publication year. Image credit: Namrata Dagli.

Overlay visualization of keyword co-occurrence, with weight attributed to TLS and scores based on average publication year, involves illustrating the interrelationships among keywords in a collection of clinical trials focusing on the impact of stress on oral health. Initially, 711 keywords were identified with a minimum occurrence threshold of one. Upon adjusting the threshold to two, 103 keywords were identified. The resulting visualization (Figure 9) included 103 keywords grouped into 15 clusters with 427 links and 644 TLS. The visualization presents the associations between keywords, with the thickness of connections indicating the frequency of their co-occurrence within the clinical trials included in the analysis. At the same time, the color denotes the average publication year of those documents, thus aiding in understanding the patterns of keyword associations and their temporal occurrence within the corpus.

The keywords within various clusters are presented in Table 4. The keywords in cluster 1 highlight different aspects of the relationship between stress and oral health. These keywords include physiological markers such as alpha-amylase and heart rate variability, psychological factors such as emotion regulation and exposure therapy, systemic connections such as the gut-brain axis, and health outcomes such as inflammation, dementia, and insomnia. Additionally, they underscore the importance of understanding the interplay between stress, psychological well-being, systemic health, and oral health outcomes for developing effective interventions and treatments. The keywords in cluster 2 cover a range of factors about the research in the field, including demographic considerations (adolescents), psychological states (anxiety, depression), dietary habits (eating), mental strain (mental stress, psychosocial stress), health conditions (obesity), stress management techniques (qigong), and broader well-being indicators (quality of life). The keywords in cluster 3 reflect various aspects of research in the field, including the examination of acute psychological stress, the influence of stress on athletes, cortisol levels, gender differences, habituation to stressors, the functioning of the hypothalamic-pituitary-adrenal axis, the process of recovery from stress, individual differences in stress reactivity, and the use of standardized stress induction tests such as the Trier social stress test. The keywords in cluster 4 reflect various aspects of the research, including psychological responses, preventive measures, intervention strategies, and physiological markers, indicating a comprehensive investigation of the complex interaction between stress and oral health. Cluster 5 indicates the research-related aspects, such as acute stress and psychosocial stressors, physiological indicators such as heart rate and blood pressure, and experimental tools such as cyber ball and virtual reality for studying stress responses. The keywords in cluster 6 suggest the following aspects of research, including potential comorbidities such as breast cancer, coping mechanisms, physiological stress response systems such as the hypothalamus-pituitary-adrenal axis, and interventions such as meditation, mindfulness, mindfulness-based stress reduction, and social support. Cluster 7 includes a range of keywords, including therapeutic interventions such as balneotherapy, psychological states such as mood, strategies for prevention, rigorous experimental designs like randomized controlled trials, relaxation techniques, biological markers such as salivary cortisol, stress reduction interventions, and various treatments aimed at addressing stress-related oral health issues. The keywords in cluster 8 indicate research on biomarkers for measuring stress effects, potential links to oral cancer, the role of exercise in mitigating stress, broader health implications, effects on immune function, physiological stress responses, and potential interventions such as yoga for stress management. Cluster 9 suggests research on psychological interventions such as cognitive behavioral therapy, physiological markers such as cortisol awakening response and diurnal cortisol, immune response indicators such as immunoglobulin A, occupational stress, and psychological distress. The keywords in cluster 10 suggest research including various stages of life, such as infancy and pregnancy, physiological mechanisms such as oxytocin regulation, genetic factors such as single-nucleotide polymorphisms, and overall well-being. This indicates a multidimensional approach to understanding the impact of stress from genetic predispositions to physiological responses and overall quality of life. Cluster 11 indicates research focussing on psychological aspects, particularly among children, including emotion, self-esteem, and resilience. Cluster 12 indicates research on the physiological effects of stress, emphasizing early life stress and its neurological implications. Cluster 13 indicates research on pain management and stress in pediatric populations. The keywords in cluster 14 delve into the gut-brain axis and probiotics, indicating interest in systemic connections. Finally, cluster 15 suggests research focused on interventions targeting stress reduction and oral health improvement.

**Table 4:** Keywords in clusters identified in co-occurrence analysis.

Clusters	MeSH keywords
Cluster 1 (13 items)	Alpha-amylase, dementia, emotion regulation, exposure therapy, gut-brain axis, heart rate variability, inflammation, insomnia, probiotic, psychological stress, salivary alpha-amylase, salivary biomarkers, sleep
Cluster 2 (10 items)	Adolescents, anxiety, depression, eating, mental stress, obesity, psychosocial stress, qigong, quality of life, stress management
Cluster 3 (9 items)	Acute psychological stress, athletes, cortisol, gender, habituation, HPA axis, recovery, stress reactivity, trier social stress test
Cluster 4 (8 items)	Acute psychosocial stress, dental anxiety, health education, health promotion, prosocial behavior, randomized controlled trials, saliva, surgery
Cluster 5 (8 items)	Acute stress, arousal, blood pressure, cyber ball, heart rate, psychosocial stressor, test, virtual reality
Cluster 6 (8 items)	Breast cancer, coping, hypothalamus-pituitary-adrenal axis, meditation, mindfulness, mindfulness meditation, mindfulness-based stress reduction, social support
Cluster 7 (8 items)	Balneotherapy, mood, prevention, RCT, relaxation, salivary cortisol, stress reduction, treatment
Cluster 8 (7 items)	Biomarkers, cancer, exercise, health, immune function, Stress response, yoga
Cluster 9 (7 items	Cognitive behavioral therapy, Cortisol awakening response, distress, diurnal cortisol, immunoglobulin A, occupational stress, psychological distress
Cluster 10 (7 items)	Infant, neonate, NICU, oxytocin, pregnancy, single nucleotide polymorphism, well-being
Cluster 11 (6 items)	Children, emotion, HIV, resilience, self-esteem, stigma
Cluster 12 (5 items)	Early life stress, EEG, glucose, memory, stress
Cluster 13 (4 items)	Child, music, pain, preterm infants
Cluster 14 (2 items)	Gut-brain axis, probiotics
Cluster 15 (1 item)	Intervention

Word Cloud

**Figure 10:**
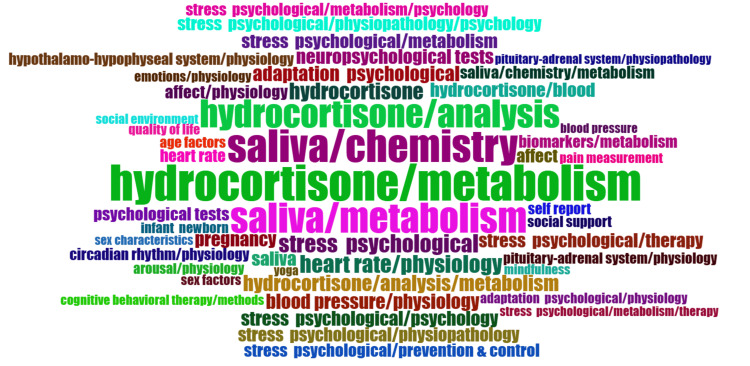
Word map showing the most frequently used keywords. Word occurrence: Square root of frequency. Image credit: Namrata Dagli.

A word cloud offers a graphical depiction of text data, where the size of each word represents its frequency or significance within the dataset. It serves as a rapid visual overview of the primary themes or subjects within the dataset, aiding researchers in pinpointing recurring terms and understanding the key areas of focus in clinical trials regarding the impact of stress on oral health. The prevalent terms in the published clinical trials on this topic included “metabolism of hydrocortisone,” “saliva chemistry,” and “hydrocortisone analysis” (Figure 10).

Analysis of Topic Trends

The analysis of topic trends across various periods highlights significant shifts in the clinical trial exploration of the effects of stress on oral health.

Before 2007, research predominantly concentrated on the correlation between various mental health conditions, such as stress, depression, and anxiety, and various physiological indicators, such as heart rate, blood pressure, circadian rhythm, as well as levels of hydrocortisone and adrenocorticotropic hormone in the blood, and this period laid the groundwork for recognizing the intricate interplay between psychological states and physiological responses.

Between 2008 and 2012, the focus shifted toward investigating psychological adaptation, employing psychological and neuropsychological tests, studying saliva composition, examining hydrocortisone levels in the blood, assessing physiological arousal, considering gender differences, exploring relaxation therapies, and investigating the relationship between affect and physiological responses such as blood pressure and heart rate. This period marked a transition toward holistically investigating stress, encompassing psychological and physiological dimensions.

From 2013 to 2015, research interests expanded to include the metabolic pathways of hydrocortisone, biomarker metabolism, saliva chemistry and metabolism, the physiology underlying the hypothalamo-hypophyseal system, and the pathophysiology of stress. These keywords collectively indicate the research focus on understanding physiological mechanisms underlying stress and its potential impacts on health.

From 2016 to 2019, there was a notable emphasis on mental health, employing cognitive behavioral therapy techniques, utilizing electroencephalography, exploring various stress management therapies, including relaxation and mindfulness techniques, and gathering self-reported data. Examining stress implications during pregnancy and in impoverished populations indicates that researchers started to recognize the importance of addressing stress not only at the individual level but also considering broader societal factors such as poverty and pregnancy.

Between 2020 and 2023, the research spotlight shifted toward exploring saliva composition, hydrocortisone levels, and the functioning of the pituitary-adrenal and hypothalamo-hypophyseal systems in response to stress. It also delved into the relationship between stress and self-concept, particularly among students, indicating a growing interest in the psychological and social dimensions of stress.

**Figure 11:**
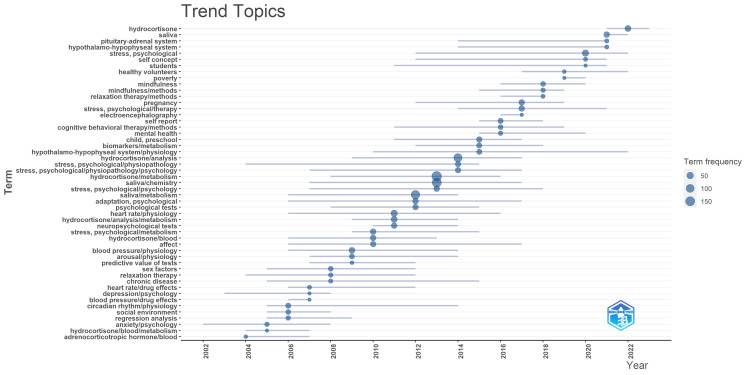
Analysis of topic trends in clinical trials published in the PubMed database on the impact of stress on oral health. Image credit: Namrata Dagli.

These shifts illustrate an evolving understanding of the impact of stress on oral health, spanning from physiological markers to psychological interventions and social contexts, offering valuable insights for clinical practice and future research directions (Figure 11).

Thematic Evolution

**Figure 12:**
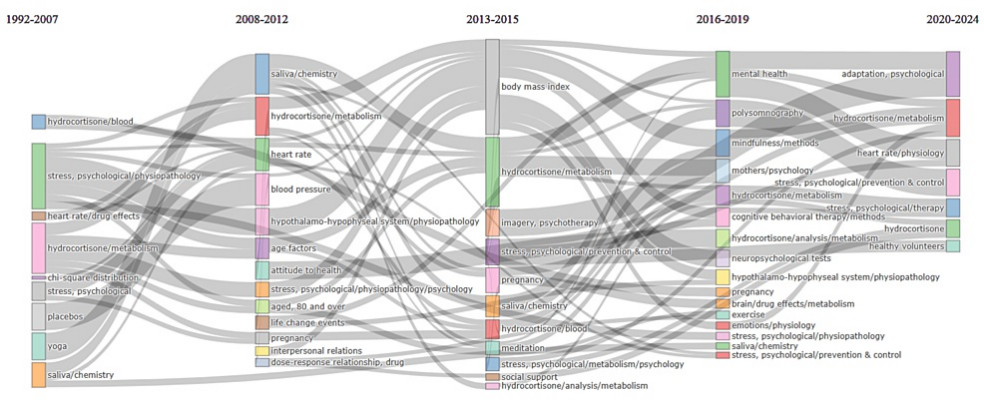
Thematic evolution analysis. Notes: Created using Biblioshiny App. Weight: Word occurrences. Clustering algorithm: Walktrap. Minimum weight index: 0.1. Minimum cluster frequency per 1,000 documents: 10. Image credit: Namrata Dagli.

The thematic evolution depicted in Figure 12 illustrates a clear progression in the focus of clinical trials investigating the impact of stress on oral health over time.

From 1992 to 2007, research predominantly centered around examining various factors such as placebo effects, age-related influences, hydrocortisone levels in the blood, drug effects on heart rate, psychometrics, affect, and the role of yoga in stress management. This period laid a foundation for understanding the physiological and psychological dimensions of stress responses.

Between 2008 and 2012, there was a notable shift toward investigating the metabolism of hydrocortisone, saliva chemistry, and physiological markers such as heart rate and blood pressure. Additionally, researchers began exploring attitudes toward health, the impact of pregnancy, interpersonal relationships, and the dose-response relationship of drugs in stress management.

From 2013 to 2015, the focus remained on hydrocortisone metabolism, but there was an increased emphasis on psychological interventions such as meditation, psychotherapy, and psychological reinforcement. Researchers also delved deeper into the physiological pathways involved in stress responses, particularly focusing on the hypothalamo-hypophyseal system and using techniques such as magnetic resonance imaging to understand stress physiology.

From 2016 to 2019, there was a significant shift toward studying the physiology of emotions and mental health, with an increased focus on cognitive behavioral therapy methods, stress pathophysiology, and neuropsychological tests. This period saw a holistic approach to stress management, incorporating techniques such as mindfulness and exercise alongside traditional therapeutic approaches.

In the most recent period, from 2020 to 2024, the focus remained on psychological adaptation and stress therapy, indicating a continued interest in understanding how individuals adapt to stressors and developing effective interventions. Hydrocortisone metabolism continued to be a significant area of investigation, along with efforts to prevent and control psychological stress, particularly in healthy volunteers.

Throughout these periods, the metabolism of hydrocortisone remained a consistent and important theme, indicating its central role in stress responses and its relevance across various aspects of stress research. Comparing the topic trends analysis and thematic evolution analysis of stress research reveals both similarities and differences in the trajectory of the investigation into the impact of stress on oral health. One consistent theme across both analyses is the sustained focus on understanding the metabolism of hydrocortisone, indicating an enduring interest in the physiological mechanisms underlying stress responses. However, while the first analysis highlights a transition from studying basic correlations between mental health conditions and physiological markers to a broader exploration of the psychological and social dimensions of stress, the second analysis emphasizes a progression from physiological markers to a more comprehensive investigation of psychological interventions and mental health outcomes. Additionally, while both analyses demonstrate a trend toward broadening the scope of research beyond purely physiological indicators, they differ slightly in the specific timing and emphasis of certain thematic areas. For instance, while the first analysis underscores a focus on psychological adaptation from 2016 to 2024, the second analysis indicates a broader exploration of mental health interventions and physiological markers during this timeframe. Nonetheless, both analyses demonstrate a clear evolution in stress research toward a more holistic understanding of stress and its multifaceted impact on health and well-being.

Thematic Map

**Figure 13:**
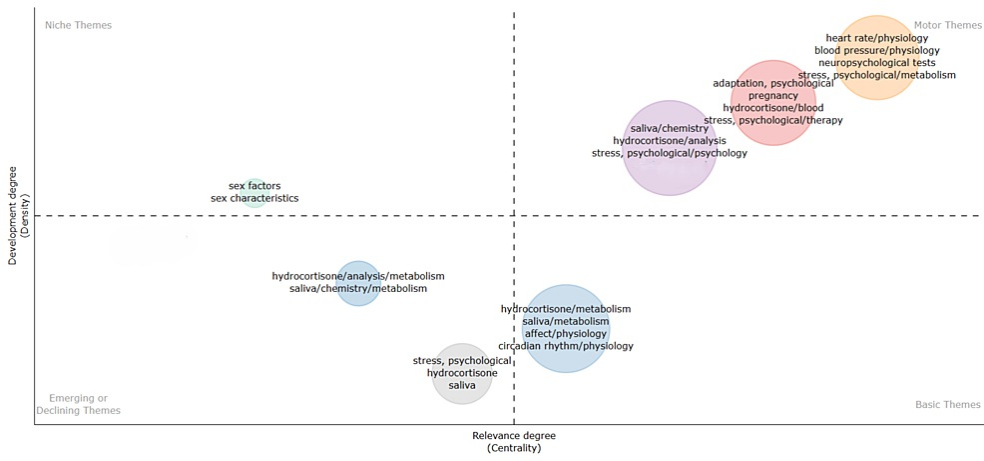
Thematic Map (created using the Biblioshiny App) based on clinical trials published in the PubMed database on the impact of stress on oral health. Image credit: Namrata Dagli.

Figure 13 is a thematic map that categorizes the themes into the following four categories according to their degree of relevance and development: motor theme, basic theme, niche themes, and emerging or declining themes. The motor themes that are the most developed and relevant to the topic include the physiology of blood pressure and heart rate, neuropsychological tests, and psychological stress metabolism. The other motor themes developed and relevant to the topic include psychological adaptation, pregnancy, psychological stress therapy, hydrocortisone analysis, and saliva chemistry.

The basic themes that are less developed but relevant to the field are the metabolism of hydrocortisone, saliva, physiology of affect, and physiology of circadian rhythm. The niche themes that are well-developed but not relevant to the topic are sex factors and sex characteristics. While they may offer valuable insights and findings, their connection to the primary topic may be tangential or indirect, making them niche areas within the broader field of study. The emerging or declining themes that are not well established include analysis of hydrocortisone metabolism and saliva chemistry and metabolism. These themes may warrant further investigation to determine their potential significance or indicate shifts in research priorities within the field. By categorizing themes based on relevance and development, researchers can gain valuable insights into where the field stands and where future efforts may be directed. Building upon the strengths of motor themes while also addressing the gaps in basic and emerging areas will be crucial for advancing knowledge and understanding in the field. Additionally, recognizing niche themes and their potential contributions can lead to interdisciplinary collaborations and further enrich the breadth of research on the topic.

Analysis of Collaboration Frequency of Countries

**Figure 14:**
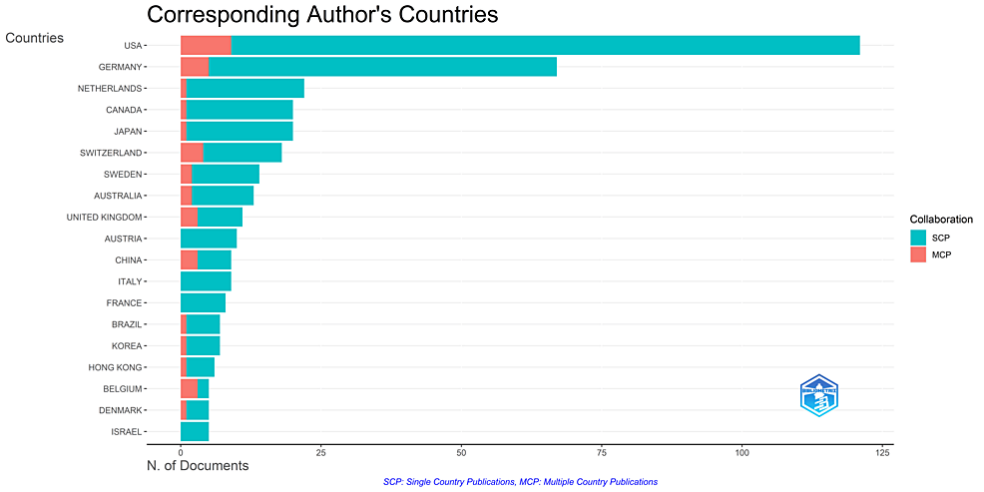
The most relevant countries are based on the number of published clinical trials on the impact of stress on oral health in the PubMed database and their collaboration pattern. Image credit: Namrata Dagli.

The analysis of the corresponding authors’ countries (Figure 14) highlights the predominant trend of conducting clinical trials within a single country. The data suggests that the United States demonstrates the highest level of collaborative efforts, followed by Germany and Switzerland. Conversely, Australia, Italy, France, and Israel appear to show no inclination toward collaboration. On the other hand, the United Kingdom, China, and Belgium demonstrate a moderate level of collaboration. At the same time, the Netherlands, Canada, Japan, Brazil, Korea, Hong Kong, and Denmark exhibit limited collaborative activity.

**Figure 15:**
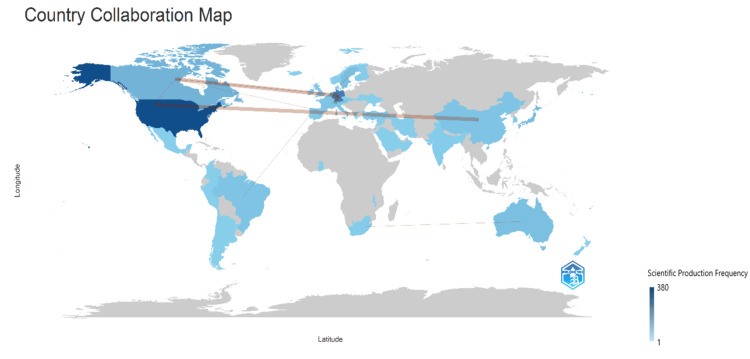
Collaboration between various countries across the world. Notes: The thickness of connecting lines indicates the frequency of collaboration between the countries they link. Image credit: Namrata Dagli.

Figure 15 illustrates the collaboration between different countries in the context of published clinical trials on the impact of stress on oral health. The intensity of the blue color reflects the number of clinical trials conducted by each country, providing insights into their research output in this field. Additionally, the thickness of the connecting lines signifies the frequency of collaboration between the countries they link. According to the figure, the highest frequency of collaboration is observed between Germany and Canada, Germany and Switzerland, and the United States and China, suggesting active engagement and cooperation in conducting clinical research across borders. Overall, this visualization offers valuable information on the extent and nature of international collaboration in clinical trials, highlighting key partnerships and areas of active engagement among countries in advancing scientific knowledge and medical research.

Discussion

The analysis of clinical trials examining the impact of stress on oral health reveals significant trends and insights. Over the years, there has been a consistent increase in publication frequency, albeit with fluctuations, suggesting sustained interest in the topic. However, the noticeable decrease in publications post-2021 indicates a potential slowdown or decline in clinical trial activity in recent years. This decline could be influenced by research priorities or shifts in research focus toward other areas. Key authors such as Kirschbaum C, Wolf OT, and Ehlert U have made substantial contributions, highlighting their influence in driving research forward. Institutions like the University of California play a leading role in producing clinical trials in this domain, indicative of collaborative efforts across academia. Psychoneuroendocrinology emerges as a pivotal journal for disseminating research findings.

Keyword analysis showcases diverse thematic clusters covering a wide range of factors, including physiological markers, psychological states, systemic connections, health outcomes, demographic considerations, stress management techniques, and intervention strategies. They highlight the importance of understanding the complex interplay between stress, psychological well-being, systemic health, and oral health outcomes. Additionally, the clusters indicate research focus areas such as psychological aspects among children, physiological effects of stress, pain management in pediatric populations, systemic connections such as the gut-brain axis, and interventions targeting stress reduction and oral health improvement. Overall, these clusters demonstrate a multidimensional approach to investigating the impact of stress on oral health, encompassing various stages of life, physiological mechanisms, genetic factors, and overall well-being.

The analysis of topic trends reveals significant shifts over different periods, spanning from basic correlations between mental health conditions and physiological indicators before 2007 to a broader exploration of psychological interventions and social contexts in recent years. Thematic evolution analysis further illustrates this progression, highlighting key themes such as physiological markers, psychological adaptation, and stress therapy. The thematic map categorizes themes into motor, basic, niche, and emerging or declining categories, providing insights into the relevance and development of different research areas within the topic. Additionally, the analysis of countries of corresponding authors reveals patterns of collaborative efforts, with the United States demonstrating the highest level of collaboration. These analyses collectively demonstrate an evolving understanding of the impact of stress on oral health, offering valuable insights for clinical practice and future research directions.

To our knowledge, no other bibliometric analysis has been published on this topic. However, one similar bibliometric analysis on the oral health-related quality of life (OHRQoL) was found [16]. The study mapped Indonesian OHRQoL research from 2018 to 2023 using Scopus and the Sinta Database, revealing an increasing number of publications and local authors, focusing on children and older populations and a predominant presence in prestigious national journals. In addition, the study recognized Universitas Indonesia, Universitas Padjajaran, and Universitas Airlangga as the foremost institutions conducting OHRQoL research in Indonesia. The International Dental and Medical Research and the Journal of the International Society of Preventive and Community Dentistry were highlighted as significant platforms for the international dissemination of research findings.

A bibliometric analysis of the relationship between oral health and stress, especially considering the lack of previous studies on this subject, is crucial for deepening our comprehension of the interplay between psychological stress and oral health consequences. By synthesizing available literature and identifying avenues for further exploration, such analysis has the potential to shape research agendas, guide clinical approaches, influence policy formulation, and drive initiatives in public health. This endeavor can lead to enhanced oral health outcomes and a better quality of life for individuals grappling with stress-related oral health challenges.

Interdisciplinary collaborations, technological innovations, and a deeper understanding of the biopsychosocial mechanisms involved have marked recent advances in the field of oral health and stress. Scholars have increasingly recognized the interconnectedness of oral health with psychological, biological, and social factors, leading to holistic approaches in research and practice. Neurobiological studies have elucidated pathways linking stress hormones to inflammatory processes in the oral cavity, while psychological interventions such as mindfulness-based stress reduction and cognitive behavioral therapy have shown promise in mitigating stress-related oral health issues [17,18]. Cognitive behavioral therapy has been proven beneficial in reducing dental phobia and dental anxiety [19,20]. Technological advancements, including wearable sensors and telehealth platforms, have facilitated personalized interventions and remote monitoring [21-24]. Microbiome research has shed light on the role of oral microbiota in mediating stress-related oral health disparities [25]. Efforts to address these disparities and translate research findings into clinical practice underscore the importance of collaborative initiatives and health equity considerations in improving outcomes for individuals affected by stress-related oral health conditions. By leveraging cutting-edge technologies, advancing our understanding of underlying mechanisms, and promoting health equity, researchers and healthcare providers can develop more effective strategies for preventing and managing stress-related oral health conditions.

**Figure 16:**
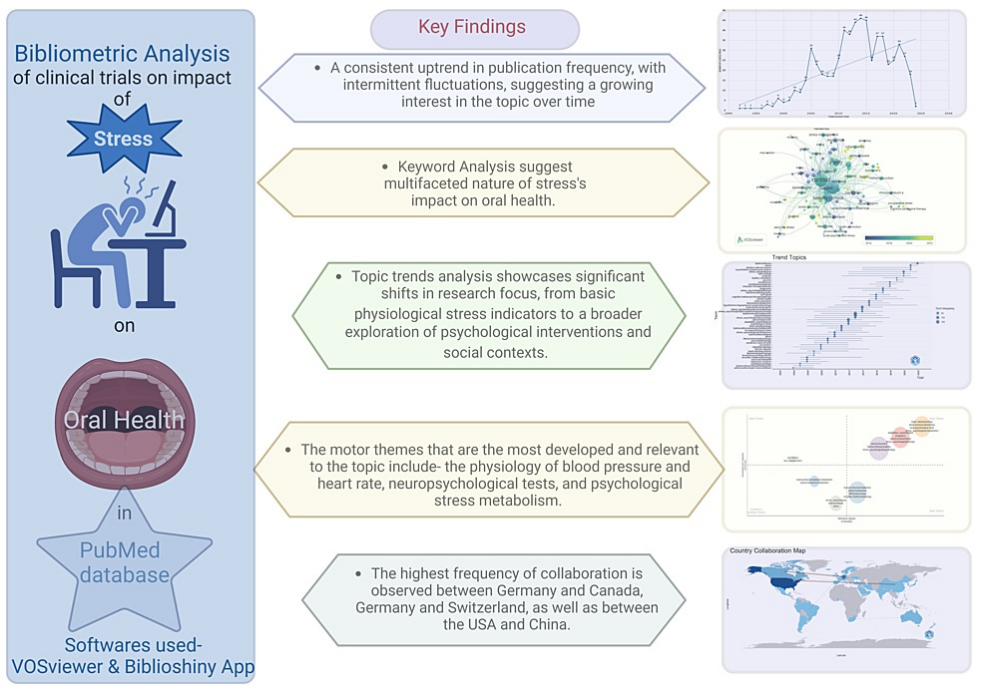
Key findings of the bibliometric analysis of clinical trials published in the PubMed database on the impact of stress on oral health. This figure has been drawn with the premium version of BioRender [15] (https://biorender.com/ accessed on March 29, 2024) with the license number TW26MUMGUB. Image credit: Namrata Dagli.

The bibliometric analysis conducted in this study offers valuable insights into the impact of stress on oral health; however, it is not without limitations. First, the study relies solely on data obtained from the PubMed database, limiting the scope of the analysis to publications indexed within this repository. While PubMed is one of the largest repositories for scientific research papers, excluding other databases may result in an incomplete representation of the literature on the topic. Additionally, the investigation is predominantly quantitative, focusing on metrics such as publication frequency and authorship patterns without assessing the quality of individual papers. As a result, the analysis may overlook important nuances or variations in research methodologies, findings, and interpretations across different studies. Despite these limitations, the study provides a comprehensive overview of research trends and influential authors and institutions in the field, offering valuable guidance for budding researchers and facilitating a deeper understanding of the subject matter. Figure 16 summarizes the key findings of this analysis, encapsulating the wealth of information derived from the bibliometric approach.

Future study recommendations

Based on the findings from clinical trials examining the impact of stress on oral health, several recommendations for future studies emerge. These include conducting longitudinal research to track the long-term effects of stress, implementing intervention studies to evaluate stress management techniques, fostering interdisciplinary collaboration to explore the complex interplay between stress and oral health, exploring novel salivary biomarkers beyond cortisol levels, investigating the influence of socioeconomic factors on the stress and oral health relationship, and integrating technology for real-time monitoring to improve oral health outcomes at the population level. By addressing these recommendations, future research can advance our understanding of the influence of stress on oral health and inform effective interventions to enhance oral health and overall well-being. Given the absence of prior bibliometric analyses on this specific topic, this study fills a notable gap in the literature, contributing to the growing body of knowledge on stress and its implications for oral health. However, future research could benefit from expanding beyond the confines of a single database and incorporating qualitative assessments to provide a more holistic understanding of the subject matter.

## Conclusions

Examining clinical trials investigating the impact of stress on oral health unveils significant trends and insights. There has been a consistent uptick over time, although fluctuations in publication frequency indicate increasing interest in the subject. Renowned authors like Kirschbaum C and Wolf OT have made notable contributions. The University of California has played a central role in generating clinical trials, while Psychoneuroendocrinology emerged as a pivotal journal for disseminating research findings in this field. Keyword analysis reveals diverse thematic clusters, reflecting the multifaceted nature of stress's impact on oral health. The analysis of topic trends in these trials shows significant shifts over different periods, evolving from fundamental correlations to broader explorations of psychological interventions and social contexts. Thematic evolution analysis further illustrates this progression. Thematic map categorizes themes into motor, basic, niche, and emerging or declining categories, offering insights into their relevance and development. Additionally, examining the countries of the corresponding authors highlights collaborative patterns, with the United States exhibiting the highest level. Together, these analyses unveil an evolving comprehension of the implications of stress on oral health, furnishing valuable insights for both clinical practice and future research endeavors.
